# Crown-Cut Endobronchial Ultrasound Guided Transbronchial Aspiration Needle: First Real-World Experiences

**DOI:** 10.3390/jcm11010163

**Published:** 2021-12-29

**Authors:** Filiz Oezkan, Woo Yul Byun, Clemens Loeffler, Udo Siebolts, Linda Diessel, Nina Lambrecht, Stephan Eisenmann

**Affiliations:** 1 Comprehensive Cancer Center, Division of Medical Oncology, The Ohio State University, Columbus, OH 43210, USA; Filiz.Oezkan@osumc.edu (F.O.); WooYul.Byun@osumc.edu (W.Y.B.); 2 Fifth Department of Medicine, University Medicine Mannheim, University of Heidelberg, 68167 Mannheim, Germany; 3 German Cancer Research Center (DKFZ), Working Group A 420, 69120 Heidelberg, Germany; 4 Department of Internal Medicine/Pneumology, University Hospital, Martin Luther University, 06120 Halle, Germany; Clemens.loeffler@uk-halle.de (C.L.); nina.lambrecht@uk-halle.de (N.L.); 5 Department of Pathology, University Hospital, Martin Luther University, 06120 Halle, Germany; Udo.Siebolts@uk-halle.de (U.S.); Linda.Diessel@uk-halle.de (L.D.)

**Keywords:** endobronchial ultrasound, crown-cut needle, 22-gauge, bronchoscopy

## Abstract

Advancements in personalized medicine have increased the demand for quantity and preservation of tissue architecture of endobronchial ultrasound-guided transbronchial needle aspiration (EBUS-TBNA) samples. These demands may be addressed by the SonoTip TopGain^®^ needle, which has a 3-point crown-cut design that contrasts with the standard single bevel design of the ViziShot 2^®^. The objective was to compare the SonoTip TopGain^®^ and ViziShot 2^®^ needles by considering biopsy sample characteristics, diagnostic accuracy, and patient safety. The primary endpoint of the study was the number of high-power fields (HPFs) in the center of the formalin-fixed paraffin-embedded cell block per sample. The lymph node with the highest probability for malignant infiltration based on size and sonographic appearance was chosen as the target lymph node for 20 patients. The same lymph node in each patient was sampled using both the ViziShot 2^®^ and SonoTip TopGain^®^ needles. The samples were measured, sliced, and analyzed by a pathologist. Sixteen patients were biopsied with both needles. Four patients could not be biopsied with the SonoTip TopGain^®^ needle since it could not penetrate cartilage or be repositioned to bypass cartilage. HPFs and sample dimensions were significantly greater in the patients where sampling with the SonoTip TopGain^®^ needle was possible (*p* = 0.007 and *p* = 0.005, respectively). Diagnostic accuracy and safety profiles were comparable. Significantly more material can be sampled using the SonoTip TopGain^®^ needle when cartilage penetration can be avoided. This improves the yield for molecular workup in the era of personalized medicine.

## 1. Introduction

Endobronchial ultrasound (EBUS) has evolved to be the first-line tool for the workup and staging of pulmonary primary tumors, metastases, and enlarged mediastinal and hilar lymph nodes in patients with sarcoidosis, tuberculosis, cancers, and lymphomas [1,2,3]. The diagnostic accuracy of EBUS for mediastinal staging has been shown to be comparable to mediastinoscopy but with the benefit of a superior safety profile [3,4,5,6,7,8,9].

Recent advancements in personalized oncology and immunotherapies requiring specialized staining, especially for programmed-death ligand 1 and mutational analysis via next generation sequencing (NGS), have increased the demand for improved quantity and preservation of tissue architecture of biopsies sampled using EBUS-TBNA. The necessary amount of tumor DNA and lymphocytes for thorough evaluation has increased beyond what is typically achieved with four passes with the traditional 22-gauge EBUS needle. The DNA requirement for NGS analysis varies by testing platform but ranges between 10 ng up to 250 ng for different NGS panels [10,11,12].

**Figure 1:**
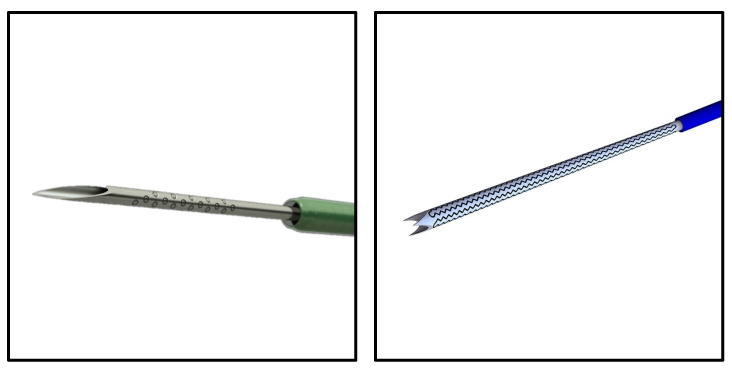
Needle Designs. The ViziShot 2^®^ (left, image credit: Olympus) is representative of the standard biopsy needle with a single bevel and point. The SonoTip TopGain^®^ (right, image credit: Medi-Globe) significantly deviates from the standard single bevel design due to its crown-cut design with three bevels and three points.

One way to address these needs is through innovative re-engineering of the EBUS-assisted transbronchial needles. New designs that modify how the biopsy sample is excised have been introduced in the recent past. A notable example is the SonoTip TopGain^®^ needle (Medi-Globe, Rohrdorf, Germany), which was recently launched in the European market. The SonoTip TopGain^®^ introduced a 3-point crown-cut needle tip design, which contrasts with the standard single bevel design of needles such as the ViziShot 2^®^ (Olympus, Tokyo, Japan) (Figure 1). The SonoTip TopGain^®^ introduces more cutting edges that would, theoretically, more evenly incise and preserve tissue and tissue architecture. However, whether the novel engineering in needle design has clinical benefits has not been fully assessed in the context of diseases leading to hilar and mediastinal lymphadenopathy. Our aim was to compare the SonoTip TopGain^®^ needle with the ViziShot 2^®^ EBUS needle by considering biopsy sample quantity, diagnostic accuracy, and patient safety.

The number of HPFs per sample was the primary endpoint of the study. This variable was chosen as the primary endpoint because the requirement for additional molecular and immunohistochemical testing is yielding more analyzable sample from each biopsy. The biopsy sample dimension was also compared because this is a general way to quantify analyzable material. The secondary endpoints are the diagnostic yield and periprocedural complications. The diagnostic yield, which was defined as whether a diagnosis could be made from a biopsy sample, was chosen as a secondary endpoint because it clarifies the diagnostic usefulness of the biopsies taken with the needles. In addition, we assessed whether the sampled material was sufficient for histopathological diagnostics and subsequent immunohistochemistry workup, two criteria important for personalized medicine.

## 2. Materials and Methods

### 2.1. Participants

The study was approved by the ethical review board of the Martin Luther University of Halle (approval number: 2019-070). Twenty patients with enlarged mediastinal and/or hilar lymph nodes requiring EBUS bronchoscopy for diagnostic workup were enrolled at the University Hospital Halle (Halle, Germany) between June and August 2020. Written informed consent was obtained from all patients. The patients’ characteristics are summarized in Table 1.

### 2.2. Needles and EBUS Procedure

We compared the 22-gauge ViziShot 2^®^ and SonoTip TopGain^®^ needles. EBUS-TBNA was performed by flexible bronchoscopy under moderate sedation with midazolam and propofol with an EBUS bronchoscope (BF-UC190F, Olympus) connected to an ultrasound scanner (EU-ME2, Olympus). A complete mediastinal and hilar lymph node staging via EBUS including sampling of all lymph nodes larger than 5 mm was performed.

The lymph node biopsies were conducted using standard procedure. The targeted lymph node was the lymph node with the highest probability for malignant infiltration based on size and sonographic appearance. Both the ViziShot 2^®^ and Sonotip TopGain^®^ needles were used to biopsy the same lymph node in each patient. Both needles were inserted into the target lymph node with the same angle, and the lymph node was sampled with 3 to 5 passes with each needle [1,13,14]. Each pass consisted of 10 to 15 needle thrusts. The ViziShot 2^®^ needle was always used first and followed by the SonoTip TopGain^®^ needle. Additional suction was not applied with either needle, and rapid on-site evaluation was not used. EBUS-TBNA samples were expelled from the needle into 5 mL of 4% buffered formalin using the internal stylet in accordance with recent guideline recommendations and were subsequently sent for cytopathological evaluation [15]. The samples were submitted to the pathology department in separate vessels labelled with the needle specifications.

### 2.3. Periprocedural Complications

Complications occurring only in the immediate procedural period were considered and assessed as previously described [16]. Complications were defined as an occurrence of bleeding requiring intervention, post-procedural infection requiring the use of antibiotics, pneumothorax, and prolonged hospitalization.

### 2.4. Cytopathological Processing

EBUS samples were processed as previously described and Papanicolaou-stained according to the standard procedure [16]. Immunohistochemistry was performed for diagnostic purposes whenever required. The length of each sample was assessed before slicing. If multiple pieces were sampled, then the lengths of each of the pieces were summed. The lengths were reported as the final dimensions. After slicing, HPFs were counted on a slide from the center of the cell block for each sample.

### 2.5. Statistical Analyses

Whether there was a large difference in the number of HPFs yielded by the two needle designs was the primary endpoint of the study. Therefore, we ensured that the study was adequately powered in the context of HPFs. The significance between the number of HPFs produced by each needle was tested using the paired, two-sided Wilcoxon signed rank test with continuity correction. With a large effect size of 0.8, type I error (*α*) probability of 0.05, and power (1 − *β*) of 0.8, the required number of patients to be adequately powered is 15. If a smaller effect size of 0.5 is considered, then the required number of patients is 36. We aimed to have a study powered to find moderately large effect sizes and thus enrolled 20 patients. Assumptions regarding the relative performance of the two needle models were not made prior to the analysis, so all statistical tests were two-sided. The two-sided Wilcoxon rank-sum test with continuity correction was used to compare the biopsy sample dimensions and number of HPFs. The paired test was used for cases when the patients were biopsied using both needles (*n* = 16). The unpaired test was used when sample dimensions from all patients were compared. The paired McNemar’s chi-squared test with Yates’ continuity correction of 0.5 was used to test the diagnostic yield, sufficiency for histopathologic diagnostics, and sufficiency for immunohistochemistry of the needle models. Only the data from patients with paired data from both needle designs were used (*n* = 16). All analyses were completed using R version 4.0.5.

## 3. Results

### 3.1. Biopsy Sample Characteristics

Lymph node samples could be collected from all 20 patients using the ViziShot 2^®^ needle but only from 16 patients using the SonoTip TopGain^®^ needle. Four samples could not be collected from four patients using the SonoTip TopGain^®^ needle because the needle could not penetrate tracheal or bronchial cartilage in the positions 11 L, 11R, and 4L.

**Figure 2:**
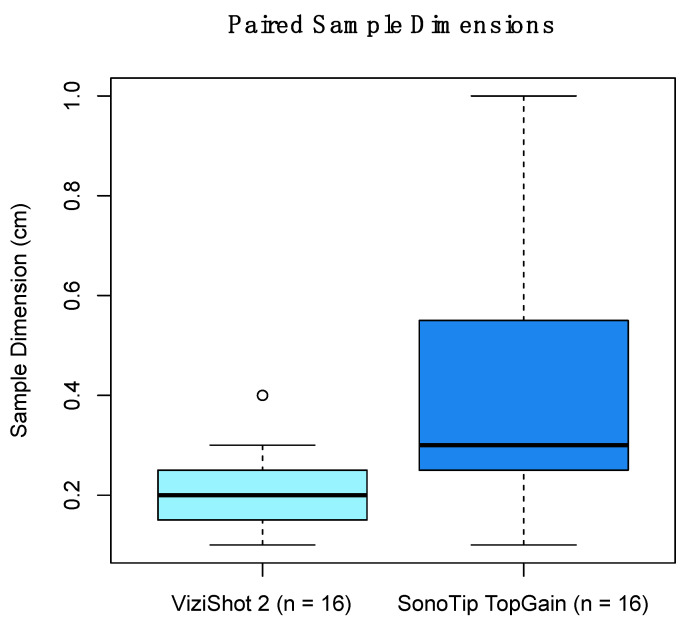
Comparison of the lymph node biopsy sample dimensions between the two needle models. Samples from 16 patients who were biopsied using both needle models are presented. The ViziShot 2^®^ yielded a mean sample dimension of 0.21 cm (SD = 0.096 cm) and the SonoTip TopGain^®^ yielded a mean sample dimension of 0.41 cm (SD = 0.24 cm), *p* = 0.007. The shaded box represents the interquartile range with the bolded horizontal line representing the median. The whiskers represent the range, excluding outliers. The outlier, which is more extreme than 1.5 times the interquartile range, is represented by the open circle.

The SonoTip TopGain^®^ needle yielded significantly larger samples than the ViziShot 2^®^ needle. When considering the 16 patients from whom samples were collected using both needle models, the ViziShot 2^®^ yielded a mean sample dimension of 0.21 cm (SD = 0.096 cm) and the SonoTip TopGain^®^ yielded a mean sample dimension of 0.41 cm (SD = 0.24 cm), *p* = 0.007 (Figure 2). If the four additional patients that were biopsied using only the ViziShot 2^®^ are included in the comparison, then the ViziShot 2^®^ yielded a mean sample dimension of 0.21 cm (SD = 0.089 cm), *p* = 0.001.

### 3.2. Histological Outcomes

**Figure 3:**
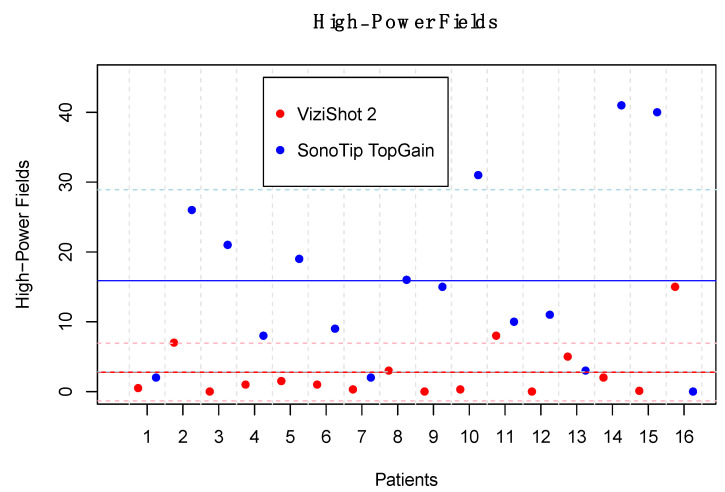
Comparison of the number of high-power fields yielded by the two needle models. Samples from 16 patients who were biopsied using both needles are presented. The dots, which are paired for each patient, represent the number of high-power fields for the respective needle model. The solid horizontal lines represent the mean number of high-power fields of each needle model (15.88 HPFs for the SonoTip TopGain^®^ needle and 2.79 HPFs for the ViziShot 2^®^ needle, *p* = 0.005). The dashed horizontal lines represent the standard deviation of the number of high-power fields of each needle model (13.04 HPFs for the SonoTip TopGain^®^ needle and 4.13 HPFs for the ViziShot 2^®^ needle).

**Figure 4:**
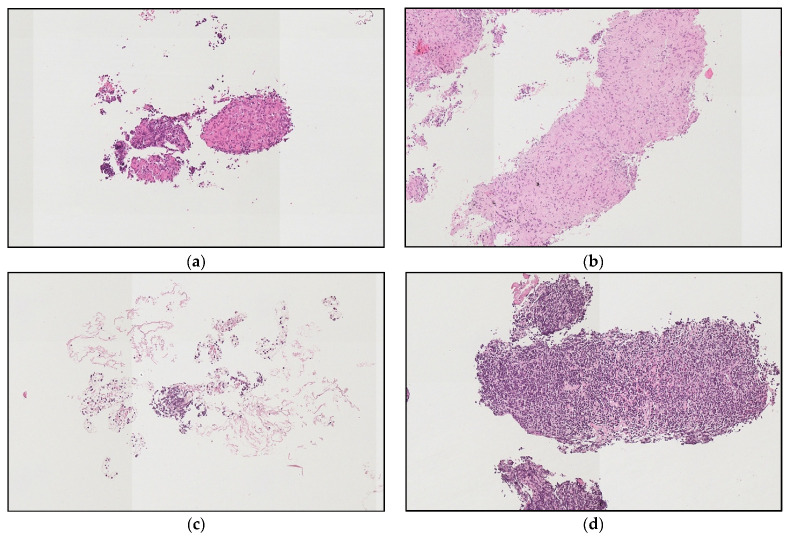
Examples of high-power fields yielded by the two needle models. (**a**,**b**) Samples from lymph node 11R of a patient with sarcoidosis. Much more sample material was obtained with the SonoTip TopGain^®^ needle (**b**) as compared to the ViziShot 2^®^ needle (**a**). (**c**,**d**) are samples from lymph node 7 of a patient with chronic lymphocytic leukemia. Material obtained with the ViziShot 2^®^ needle (**c**) was insufficient for histopathologic diagnosis, while sufficient when sampled with the SonoTip TopGain^®^ needle (**d**). Hematoxylin and eosin staining; magnification for all images was 140×.

On average, samples biopsied with the SonoTip TopGain^®^ needle yielded significantly more HPFs than samples taken with the ViziShot 2^®^ needle for the 16 patients from whom samples were collected with both needles. Samples taken with the ViziShot 2^®^ needle yielded an average of 2.79 HPFs (SD = 4.13 HPFs) while the SonoTip TopGain^®^ needle yielded an average of 15.88 HPFs (SD = 13.04 HPFs), *p* = 0.005 (Figure 3 and Figure 4). Biopsy samples obtained with the ViziShot 2^®^ needle showed a positive trend towards being more sufficient for histopathological diagnostics and immunohistochemistry (*p* = 0.153 and *p* = 0.080, respectively; Table 2).

The diagnoses included regular lymph node, fibrotic lymph node, sarcoidosis, tuberculosis, coal worker’s pneumoconiosis, renal cell carcinoma, colorectal cancer, non-small cell lung cancer, small cell lung cancer, esophago-gastric cancer, chronic lymphocytic leukemia, and Hodgkin’s lymphoma (Table 1). Of note, patients with Hodgkin’s lymphoma and chronic lymphocytic leukemia could only be diagnosed after sampling with the SonoTip TopGain^®^ needle.

### 3.3. Complications

No complications occurred in the peri- and postprocedural time.

## 4. Discussion

The main question of whether the 3-point crown-cut needle tip design of the SonoTip TopGain^®^ has potential benefits to personalized medicine has been answered by considering the primary and secondary endpoints of this study.

The primary endpoint was the number of HPFs. The results demonstrated that there were significantly more HPFs for histological use when samples could be collected using the SonoTip TopGain^®^ needle. In addition, the sample dimensions were significantly greater. This strongly supports that using the SonoTip TopGain^®^ needle is more favorable for the higher tissue yields required for advancements in personalized medicine since more tissue implies more DNA and lymphocytes.

However, lymph node samples could not be collected using the SonoTip TopGain^®^ in four cases because the needle could not penetrate tracheal or bronchial cartilage in the positions 11L, 11R, and 4L. Difficulty in reaching such nodal stations have been previously reported [17]. This links the needle’s efficacy to lymph node location and anatomy. Thus, whether using the SonoTip TopGain^®^ is appropriate is determined by the position of the lymph node and nearby cartilage, which is a major shortcoming compared to the standard ViziShot 2^®^ needle. These results suggest a protocol to first analyze imaging to determine whether cartilage is an obstacle, then choose the more appropriate needle to use.

The secondary endpoint included the diagnostic yield and periprocedural complications. The diagnostic yield, along with the capacity for histopathological diagnostics and immunohistochemistry, were not significantly different between the needle models. The lack of severe complications using either needle suggests that safety is virtually equal. Therefore, both needles have similar diagnostic and clinical value. Future assays that require more biopsied material have a strong potential to make crown-cut needle designs, such as the SonoTip TopGain^®^, have more diagnostic value.

### 4.1. Current Limitations

This study has several limitations. The largest limitation is that the study enrolled 20 patients with only 16 patients yielding the paired data. Although this number of patients yielded adequate power to explore the endpoints, the relative low sample size makes comparisons of observations with small effect sizes difficult and restricts the scope of the study. Discerning differences in diagnostic yield, sufficiency for histopathological diagnostics, and sufficiency for immunohistochemistry may be limited by the sample size. The observations for each diagnosis are few, and thus, any benefits of using the SonoTip TopGain^®^ over the ViziShot 2^®^ in the context of specific diseases are unclear. Another limitation is that this is a single-center study. This may limit generalizability. Lastly, the needle model was not blinded to the pathologist analyzing the sample.

### 4.2. Future Directions

Although general efficacy of the SonoTip TopGain^®^ needle was demonstrated when compared to the standard ViziShot 2^®^ needle, further elucidation of the clinical usefulness of the new needle design in the context of specific diagnoses would be fruitful since earlier diagnoses and prognoses are domains where advancements in personalized medicine have the most potential. One such context are hematologic disorders.

Even though diagnostic yields of EBUS-TBNA samples of lung cancer patients are very high (>90%), diagnostic yields of both Hodgkin and non-Hodgkin lymphoma patients’ lymph node samples have been reported to be as low as 68.7% when sampled with a 21- or 22-gauge conventional EBUS needle [1,16,18,19]. The definitive diagnosis of both a Hodgkin’s and non-Hodgkin’s lymphoma requires the evaluation of the tissue architecture, cell morphology, and immunophenotype and generally requires more tissue than is needed for the diagnosis of solid tumors [1]. In this study, patients with Hodgkin’s lymphoma and chronic lymphocytic leukemia could only be diagnosed after sampling with the 22 G SonoTip TopGain^®^ needle, hinting that the additional tissue dimensions and HPFs have valid clinical value. For rare diseases such as pneumoconiosis, the diagnostic yield data has not yet been reported. Therefore, further investigation of the diagnostic yield in these diseases with larger patient populations will, in our view, provide evidence that the SonoTip TopGain^®^ needle offers an advantage over the ViziShot 2^®^ needle for higher diagnostic yield.

Furthermore, studies on how the new needle design fares against other novel approaches to meeting the demands of personalized medicine will be valuable. One such approach is the recently reported transbronchial mediastinal cryobiopsy. Recent studies support that cryobiopsy yields larger amounts of intact tissue and has improved diagnostic yield when compared with EBUS-TBNA [20,21,22]. However, how this new technique compares with the SonoTip TopGain^®^ needle has not been studied.

## 5. Conclusions

This study compared the novel crown-cut SonoTip TopGain^®^ needle against the standard single bevel ViziShot 2^®^ needle and showed that the SonoTip TopGain^®^ needle yields significantly more tissue and HPFs but did not show significant differences in diagnostic yield and safety. The SonoTip TopGain^®^ needle could not easily penetrate cartilage, which poses a new engineering problem. This information validates the crown-cut needle design as a way to facilitate adaptation of new developments in personalized medicine and suggests that novel engineering that improves how biopsy samples are collected can be a valuable approach to future patient care.

## Figures and Tables

**Table 1:** Patient Characteristics and Diagnoses.

Study Population (*n* = 20)			
**Age**—mean years (range)	56.6 (23–76)		
**Smokers**	11/20		
**Pack Years of Smokers**—mean years (range)	28 (7–40)		
	**Total**	**Sampled with** **ViziShot 2^®^ (*n* = 20)**	**Sampled with** **SonoTip TopGain^®^ (*n* = 16)**
**EBUS/EUS-B**	3/20	3/20	3/16
**Lymph Node Position/Site**			
2R	1	1	1
2L	1	1	1
4R	7	7	7
4L	2	2	1
7	4	4	4
11R	4	4	2
11L	1	1	0
**Size of Sampled Node**—mean millimeter (range)	16.45 (9–30)	16.45 (9–30)	18 (9–30)

**Prior Malignant Diagnosis** (yes/no)	11/9	11/9	10/6
**Diagnosis**			
Regular lymph node	5/20	5/20	3/16
Fibrotic lymph node	1/20	0/20	1/16
Sarcoidosis	2/20	2/20	1/16
Tuberculosis	1/20	1/20	1/16
Coal worker’s pneumoconiosis	1/20	0/20	1/16
Renal cell carcinoma	1/20	1/20	1/16
Colorectal cancer	2/20	2/20	2/16
NSCLC	1/20	1/20	1/16
SCLC	2/20	1/20	2/16
CLL	1/20	0/20	1/16
Hodgkin’s lymphoma	1/20	0/20	1/16
Esophago-gastric cancer	1/20	0/20	1/16
Hypopharyngeal cancer	1/20	1/20	0/16

EBUS = endobronchial ultrasound; EUS-B = endoscopic ultrasound; CLL = chronic lymphocytic leukemia; NSCLC = non-small cell lung cancer; SCLC = small cell lung cancer.

**Table 2:** Comparison of the number of patients out of 16 who were biopsied using the SonoTip TopGain^®^ and the ViziShot 2^®^ needles regarding sufficiency of the biopsies for histopathological diagnostics, immunohistochemistry, and diagnostic yield. There were no significant differences between the two needles for all criteria (*p* = 0.153, *p* = 0.080, and *p* = 0.080, respectively).

Sufficiency for Histopathological Diagnostics (*n* = 16)		SonoTip TopGain^®^	
		Sufficient	Insufficient
**ViziShot 2^®^**	**Sufficient**	9	1
	**Insufficient**	5	1
**Sufficiency for Immunohistochemistry (*n* = 16)**		**SonoTip TopGain^®^**	
		**Sufficient**	**Insufficient**
**ViziShot 2^®^**	**Sufficient**	12	0
	**Insufficient**	4	0
**Diagnostic Yield (*n* = 16)**		**SonoTip TopGain^®^**	
		**Diagnosed**	**Not Diagnosed**
**ViziShot 2^®^**	**Diagnosed**	12	0
	**Not Diagnosed**	4	0

## Data Availability

All data generated or analyzed during this study are included in this article. Further enquiries can be directed to the corresponding author.
